# Genomic Positional Dissection of RNA Editomes in Tumor and Normal Samples

**DOI:** 10.3389/fgene.2019.00211

**Published:** 2019-03-20

**Authors:** Michael Chigaev, Hui Yu, David C. Samuels, Quanhu Sheng, Olufunmilola Oyebamiji, Scott Ness, Wei Yue, Ying-yong Zhao, Yan Guo

**Affiliations:** ^1^Department of Internal Medicine, The University of New Mexico, Albuquerque, NM, United States; ^2^Department of Molecular Physiology and Biophysics, Vanderbilt Genetics Institute, Vanderbilt University School of Medicine, Nashville, TN, United States; ^3^Department of Biostatistics, Vanderbilt University Medical Center, Nashville, TN, United States; ^4^Key Laboratory of Resource Biology and Biotechnology in Western China, School of Life Sciences, Northwest University, Xi’an, China

**Keywords:** RNA editing, A-to-I, adenosine to inosine, cancer, non-coding RNAs, TCGA, GTEx

## Abstract

RNA editing is phenomenon that occurs in both protein coding and non-coding RNAs. Increasing evidence have shown that adenosine-to-inosine RNA editing can potentially rendering substantial functional effects throughout the genome. Using RNA editing datasets from two large consortiums: The Cancer Genome Atlas (TCGA) and Genotype-Tissue Expression (GTEx) project, we quantitatively analyzed human genome-wide RNA editing events derived from tumor or normal tissues. Generally, a common RNA editing site tends to have a higher editing level in tumors as compared to normal samples. Of the 14 tumor-normal-paired cancer types examined, Eleven of the 14 cancers tested had overall increased RNA editing levels in the tumors. The editomes in cancer or normal tissues were dissected by genomic locations, and significant RNA editing locational difference was found between cancerous and healthy subjects. Additionally, our results indicated a significant correlation between the RNA editing rate and the gene density across chromosomes, highlighted hyper RNA editing clusters through visualization of running RNA editing rates along chromosomes, and identified hyper RNA edited genes (protein-coding genes, lincRNAs, and pseudogenes) that embody a large portion of cancer prognostic predictors. This study reinforces the potential functional effects of RNA editing in protein-coding genes, and also makes a strong foundation for further exploration of RNA editing’s roles in non-coding regions.

## Introduction

RNA editing is the molecular process by which ribonucleic acid (RNA) molecule’s nucleotide sequences are modified enzymatically after RNA has been generated by RNA polymerase. There are two types of canonical RNA editing: adenine-to-inosine (A-to-I) and cytosine-to-uracil (C-to-U). The mechanisms of these two types of RNA editing have been thoroughly studied. The A-to-I editing is mediated by adenosine deaminase acting on RNA (ADAR) (Bass and Weintraub, 1987), and C-to-U editing happens when cytidine deaminase deaminates a cytidine base into a uridine base (Gott and Emeson, 2000). A-to-I RNA editing accounts for >95% of all reported RNA editing events and over four hundred million adenosine sites in the human genome are presumed to be subject to A-to-I editing (Bazak et al., 2014; Picardi et al., 2017). The mapping of whole-genome RNA editing events leads to the elucidation of inosinomes (Picardi et al., 2015) or editomes (Diroma et al., 2017).

Most well characterized functional editing sites are found in transcripts for neuronal transporters and channel proteins in the brain (Meier et al., 2016). Recent advances in RNA editing research have revealed functional effects of A-to-I RNA editing in extended contexts, especially tumors. Peng et al. (2018), demonstrated experimentally that non-synonymous A-to-I RNA editing can result in alternative protein sequences by modifying amino acids in cancer, and may subsequently affect drug sensitivity regulation (Han et al., 2015). Furthermore, increased editing activity has been associated with poor cancer prognosis (Paz-Yaacov et al., 2015). A comprehensive review of the roles of RNA editing in human cancers was published recently (Qian et al., 2018).

Given a large amount of paired DNA-RNA sequencing data accumulated over the last decade, it became feasible to detect RNA-DNA Difference from direct comparison of DNA and RNA nucleotide sequences, and millions of RDDs have been documented in RNA editing databases, such as REDIportal (Picardi et al., 2017). REDIportal was based on the Genotype-Tissue Expression (GTEx) data (Lonsdale et al., 2013) which has been used for comprehensive RNA editing analysis (Tan et al., 2017). Other than studying the occurrence of RNA editing events (qualitative identification), there is an increasing interest in examining the level of RNA editing, especially in experiments that involve multiple conditions (quantitative comparison). Technically speaking, the “RNA editing level” of an RNA sample can be calculated as the percentage of edited bases at an RNA editing site, which is an index specific to a particular biological condition. For instance, JACUSA (Piechotta et al., 2017) was empowered to compare RNA editing level across different experimental conditions, thus detecting RNA-RNA differences. In a study of Wood-decaying fungi, JACUSA was employed to compare RNA editing levels across varied culture conditions and it helped confirm the more prominent impact of culture conditions than host wood species (Peng et al., 2018). In analogy to the pervasive differential expression analyses in transcriptome studies, the rising editome studies will soon be pivoting on differential editing analyses.

Abundant high-throughput sequencing data of human cancers enable us to study the differential RNA editing level in tumor samples relative to normal. Here, in various perspectives, we performed RNA editing level analyses on 6,236 samples from 14 cancers from TCGA, aiming to obtain global views on RNA editing levels across different sample sources (tumor vs. health), genomic locations (exonic, intronic, and intergenic), and gene features (protein-coding genes, lincRNAs, and pseudogenes). Because of the dominance of A-to-I RNA editing, our study primarily focused on the canonical A-to-I editing type.

## Materials and Methods

We obtained 230,079 RNA editing events across 107,049 genomic positions from a previous RNA editing study by Han et al. (2015). These RNA editing sites were identified from 6,236 samples of 14 cancers from TCGA. These samples were collected from 5,672 subjects, 544 of which had paired tumor and normal samples. The RNA editing detection methodology and filters were described in detail by Han et al. (2015). The RNA editing sites obtained from Han et al. (2015) study were the sites passed their quality control measures, and used to generate their results. Our study is an extension of Han et al. (2015) study, while verifying some of the previous conclusions, we focused more on the non-coding regions.

The level and rate of RNA editing were computed using the following formulas:

$$\mathrm{RNA editing level}=\frac{\mathrm{number of reads supporting edited allele}}{\mathrm{total number of reads at a locus}}$$

$$\mathrm{RNA editing rate}\;(\mathrm{of a gene or a chromosome})=\frac{\mathrm{number of RNA editing events within the bounded region}}{\mathrm{length of the bounded region}/1000}$$

Gene edit frequency was defined as number of RNA edit within the gene divided by the gene length. Hyper RNA edited genes were identified by rank the gene edit frequency from large to small.

Furthermore, 4.66 million known RNA editing events from database REDiportal (Picardi et al., 2017) were downloaded and summarized for comparison purpose. Built primarily upon a different source “GTEx project.” The detailed RNA editing identification method is described in REDiportal publication (Picardi et al., 2017).

In the present study, RNA editing in chromosome 1–22, X and Y were studied thoroughly, while the mitochondrial genome was omitted. Annotation of the RNA editing positions was done using ANNOVAR (Wang et al., 2010). Wilcoxon Rank Sum test was used to compare the RNA editing level between tumor and normal. A Benjamini Hochberg adjusted *p*-value <0.05 was used as the significant threshold.

## Results

### Positional Dissection of RNA Editing Events

**FIGURE 1:**
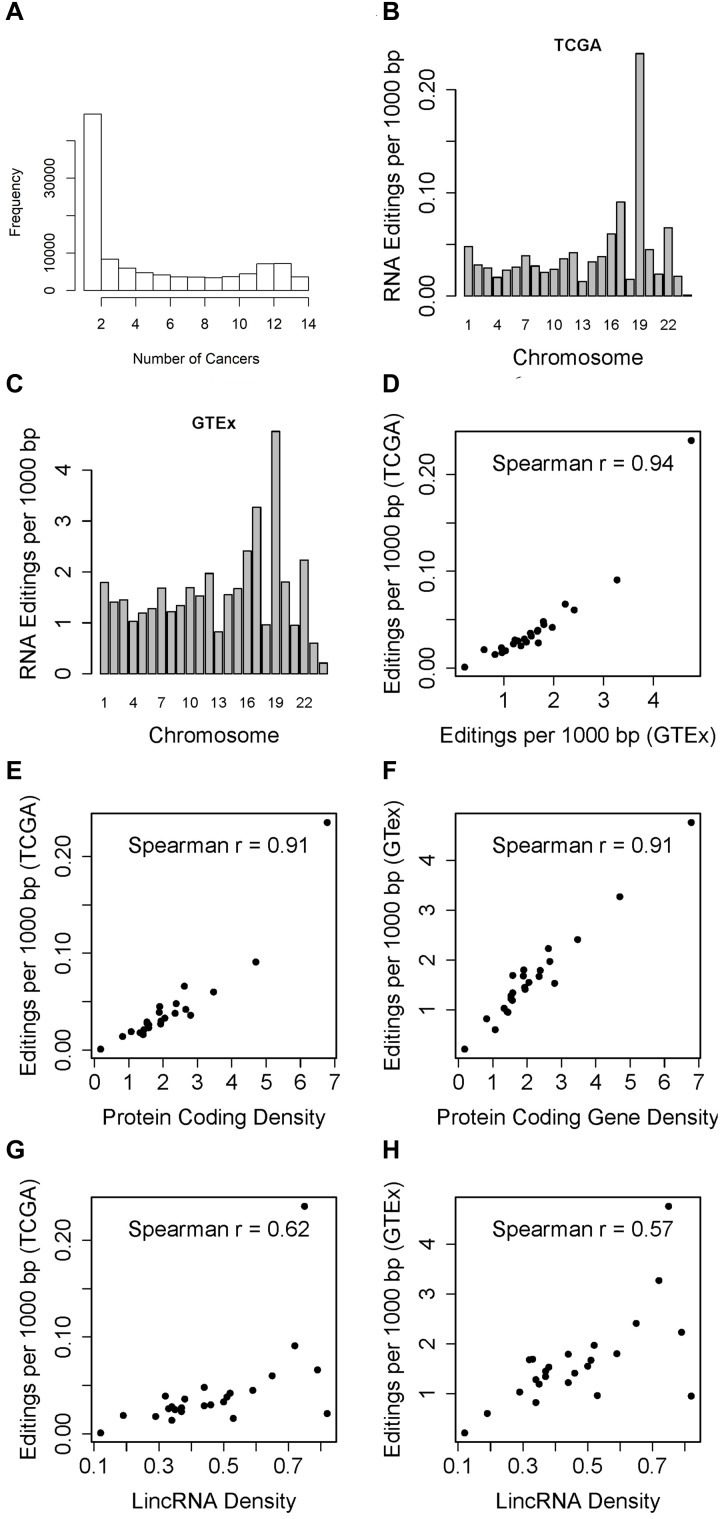
**(A)** Histogram which displays the number of times RNA editing positions have been observed in the 14 cancer types. Majority of the RNA editing positions were observed in one cancer type. **(B)** Barplot which shows the RNA editing rate in TCGA (RNA editing events per 1000 bp) by chromosome. The RNA editing rate is adjusted for the chromosome length. Chromosome 19 had the highest RNA editing rate. **(C)** Barplot which shows the RNA editing rate in GTEx chromosome. The RNA editing rate is adjusted for the chromosome length. Similar to GTEx results, chromosome 19 had the highest RNA editing rate. **(D)** Scatter plot of overall RNA editing rates by chromosome between TCGA and GTEx by chromosome. **(E)** Scatter plot of TCGA RNA editing rate and gene density by chromosome. **(F)** Scatter plot of GTEx RNA editing rate and gene density by chromosome. **(G)** Scatter plot of TCGA RNA editing rate and lincRNA density by chromosome. **(H)** Scatter plot of GTEx RNA editing rate and lincRNA density by chromosome.

In the TCGA data, across all 14 cancer types, 230,079 RNA editing events across 107,049 genomic positions were identified (Supplementary Table S1), as compared to 4.66 million editing sites documented in REDIportal. Of the 107,049 RNA edited positions, 32.3% occurred in one cancers, with only 3,654 (3.4%) RNA editing events shared across all 14 types of cancer examined (Figure 1A). These results suggest that one third of the RNA editing sites detected in TCGA are cancer specific. To further valid this, we used average RNA editing level >10%, >25%, and >50% as RNA editing site cutoffs and repeated the analysis. Similar cancer specificity were observed (Supplementary Figure S1). TCGA’s cancers were tissue specific, and tissue specific RNA editing sites has been found in normal subjects in GTEx (Picardi et al., 2017). This suggests the same tissue specificity of RNA editing in cancer subjects. 95.6% of these RNA editing positions are in Alu regions. There is no correlation between sample size and number of RNA editing events identified (Spearman *r* = −0.04, *p* = 0.9, Supplementary Figure S2).

When examining the number of RNA editing events by chromosome, we immediately notice an interesting pattern, i.e., after adjusting for chromosome length, chromosome 19 always had the highest RNA editing rate measured by number of RNA editing events per 1000 base pairs in TCGA (Figure 1B and Supplementary Table S2). Likewise, GTEx data demonstrated the similar high RNA editing rate for chromosome 19 (Figure 1C). The RNA editing rates between TCGA and GTEx by chromosome are highly correlated (Figure 1D, Spearman *r* = 0.94). Upon further examination, we found that when comparing the abundance of RNA editing events across chromosomes, it is not the chromosome length but the gene density that should be accounted for. Gene density can be represented as the exome percentage per chromosome calculated as sum of all exon length within a chromosome divided by the chromosome length (Guo et al., 2017). We computed both protein-coding gene and lincRNA gene densities. Protein coding gene density is strongly correlated with the overall RNA editing rate in TCGA (Figure 1E, Spearman *r* = 0.91), and with that from GTEx as well (Figure 1F, Spearman *r* = 0.91). The correlations between lincRNA gene density and RNA editing rates are relatively weaker, with Spearman correlation coefficients 0.62 for TCGA and 0.57 for GTEx (Figure 1G,H). Chromosome 19 is visibly the outlier in the scatter plots with the highest RNA editing rate.

**FIGURE 2:**
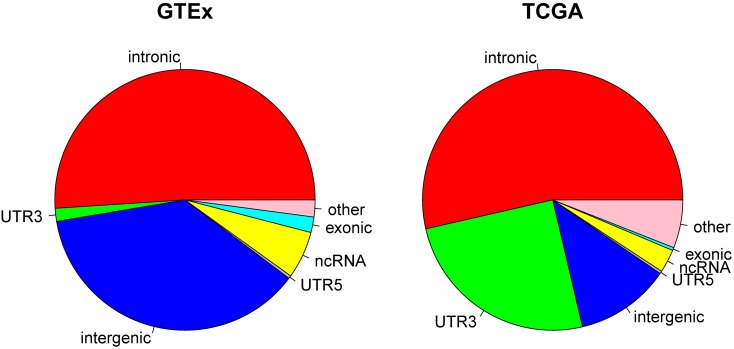
Pie charts display the distribution of RNA editing positions by genomic location categories. Most of RNA editing happens in intronic regions. Large proportional differences can be observed between GTEx and TCGA.

Because current sequencing data mostly target protein coding regions, it is not surprising that a majority of the RNA editing events reported from GTEx and TCGA were found in or close to exome regions. In TCGA, we observed that 53.58% of the RNA editing events occurred in intronic regions, followed by 3′UTR (25.10%). Non-coding RNA accounted for 2.89%, and intergenic regions accounted for only 11.77% of all RNA editing in TCGA. In GTEx, 51.03% of RNA editing occurred in introns and 1.61% occurred in 3′UTRs. Non-coding RNA accounted for 5.89%, and intergenic regions accounted for 37.08% of all RNA editing in GTEx (Figure 2). The distribution of each individual cancer can be found in Supplementary Figure S3. The prominent presence of 3′UTR and intronic RNA editing is due to the high density of Alu elements in such regions (Guo et al., 2018). There is a notable RNA editing proportional difference between GTEx and TCGA. For example, the 3′UTR proportion in TCGA (25.01%) is significantly (Chi-square *p* < 0.0001) larger than GTEx (1.61%). The overall Chi-square tests between TCGA and GTEx was also significant with *p* < 0.0001. Such a difference might be related to the sample source difference, as TCGA subjects were all cancer patients and the normal samples were adjacent normal tissues whereas GTEx subjects were all considered healthy individuals.

### RNA Editing Level: Tumor vs. Normal

**FIGURE 3:**
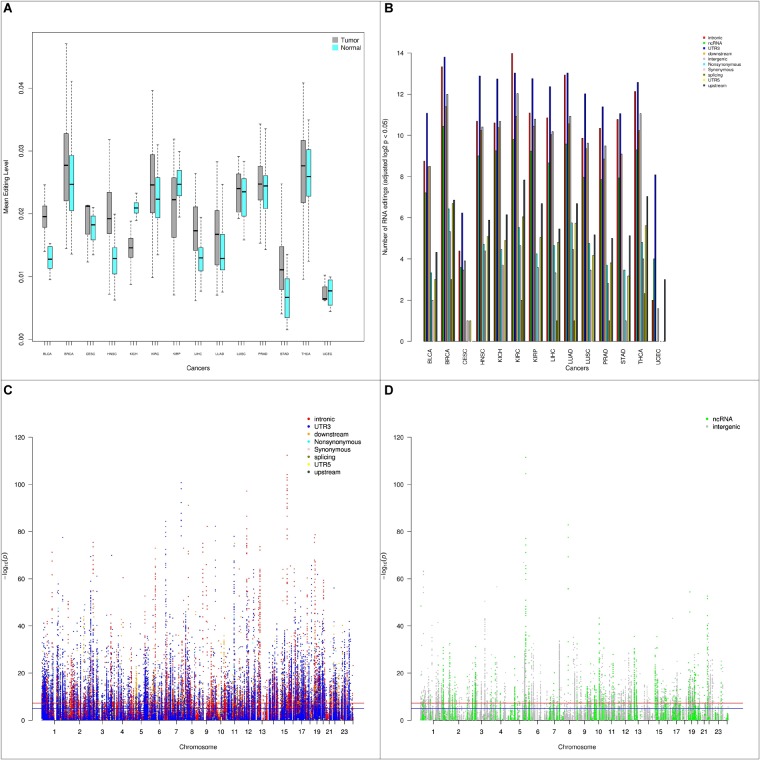
**(A)** Box plots depicting the average RNA editing levels between paired tumor and normal samples by cancer type. Majority of the cancer types have higher RNA editing levels in tumor than normal. **(B)** Barplot depicting number of RNA editing events with *p* < 0.05 between tumor and normal by cancer type and by genomic location categories. **(C)** Manhattan plot of genome-wide differential RNA editing level results for protein-coding genes. **(D)** Manhattan plot of genome-wide differential RNA editing level results for lincRNA and pseudogenes.

We examined the RNA editing level between tumor and normal samples. Using paired tumor and normal samples from the same patients (544 pairs), we conducted paired *t*-tests to detect any significant RNA editing level differences. Stratifying by cancer types, of the 14 types of cancer with tumor-normal pairs, 11 types of cancers had higher RNA editing levels in tumor and 3 types of cancer (KICH, KIRP, UCEC) had higher RNA editing levels in normal (Figure 3A). UCEC has a small sample size of four tumor-normal pairs, thus it is statistically less relevant. Both KICH and KIRP are kidney related cancer. The higher RNA editing levels in normal could be kidney-specific, however, the third type of kidney cancer KIRC had higher RNA editing levels in tumor.

Next, we examined RNA editing levels between tumor and normal samples per RNA editing site. The RNA editing events were categorized based on their genomic location: intronic, exonic, 3′-UTR, 5′-UTR, upstream, downstream, splicing, ncRNA, and intergenic. As expected, the number of significantly differentially edited positions are dominated by intronic and 3′-UTR RNA editing events and influenced by the sample size in each cancer type (Table 1 and Figure 3B). The genome-wide positional and significance information of these differentially edited positions are presented in Manhattan plots (Figure 3C,D and Supplementary Figure S4). The separate Manhattan plot for non-coding RNA and intergenic regions were generated (Figure 3D). The detailed differential RNA editing analyses are available in Supplementary Table S3. Functional analyses were conducted using David (Huang et al., 2009). Of the 1979 unique function categories identified (Supplementary Table S4), 375 categories have regulatory effects on biological processes or factors such as gene expression, metabolic process, and TOR signaling. The function categories overlapping statistics are presented in Supplementary Table S5.

**Table 1:** Differential edited positions by cancer and genomic position categories.

Cancer	Sample size	downstream	Intergenic	Intronic	ncRNA	Non-synonymous	Splicing	Synonymous	Upstream	UTR3	UTR5
BLCA	271	355	360	430	148	10	0	4	20	2162	8
BRCA	942	2684	4077	10323	1390	86	8	40	116	14217	103
CESC	199	11	15	21	12	1	0	2	0	75	2
HNSC	468	1209	1360	1645	511	26	1	21	59	7555	34
KICH	91	1322	1639	1559	609	22	1	13	71	6828	30
KIRC	515	1949	4170	16069	892	46	4	25	229	8342	66
KIRP	228	1398	1764	2180	602	19	1	12	103	6894	33
LIHC	250	1045	1158	1842	406	25	2	10	44	5272	28
LUAD	546	1516	1953	7776	767	54	2	22	103	8349	53
LUSC	237	657	786	928	249	27	0	11	36	4146	18
PRAD	426	463	717	1298	232	13	2	7	32	2670	14
STAD	318	547	1	1752	244	11	1	2	35	2124	9
THCA	557	1205	2121	4493	627	28	5	16	131	6087	49
UCEC	320	1	3	4	16	0	0	1	8	271	1

### Hyper RNA Editing

Using RNA editing data from both GTEx and TCGA, we sought to identify hyper RNA editing genes. The top 10 hyper RNA edited genes from TCGA and GTEx data are displayed in Table 2. For TCGA, the top 10 hyper RNA editing genes were all protein-coding genes. According to The Human Protein Atlas (Atlas), all ten genes but one were confirmed to have important prognostic value. For example, renal cancer patients with higher TLCD2 expression were found to have worse survival time (Atlas); upregulated SPN expression was found to associate with better survival in endometrial, breast and melanoma cancers (Atlas). Notably, 3 of the 10 hyper RNA edited genes (CYCs, NDUFB1 and COQ4) are involved in the electron transfer chain in mitochondria. For GTEx, the top 10 hyper editing genes include 5 protein-coding genes, 2 pseudogenes, and 3 lincRNAs. Three out of the 5 protein-coding genes were found to have prognostic value (Atlas). The top 10 hyper editing genes by cancer are presented in Supplementary Table S6.

**Table 2:** Hyper RNA editing genes.

Source	Gene	Gene type	Editing events	Editing events per 1000 bp	Cancer prognostic
GTEx	GTF2IP23	Pseudogene	454	199.65	None
	IPO5P1	Pseudogene	427	147.90	None
	LINGO3	Protein coding	253	112.90	None
	HTR1F	Protein coding	282	89.81	None
	GS1-124K5.11	lincRNA	1414	85.51	None
	C19orf71	Protein coding	380	77.92	Renal↑, pancreatic↑, head and neck↑
	LINC00354	lincRNA	89	74.66	None
	THRIL	lincRNA	142	71.83	None
	AMZ2	protein coding	634	66.10	liver↓, renal↓
	HNRNPA1L2	Protein coding	194	64.43	Breast↑, pancreatic↑
TCGA	TLCD2	Protein coding	121	45.34	Renal↓
	SPN	Protein coding	252	31.95	Endometrial↑, breast↑, melanoma↑
	CYCS	Protein coding	135	25.61	Renal↑, head, and neck↓
	PDDC1	Protein coding	230	22.40	Renal↑, liver↓
	NDUFB1	Protein coding	118	20.36	Renal↑
	COQ4	Protein coding	217	18.81	Endometrial↑, renal↑, cervical↑
	MRI1	Protein coding	148	15.18	None
	FAM98C	Protein coding	85	14.28	Ovarian↓, urothelial↑
	KAT8	Protein coding	217	13.87	Lung↑
	CCDC84	Protein coding	243	13.77	Renal↑

**FIGURE 4:**
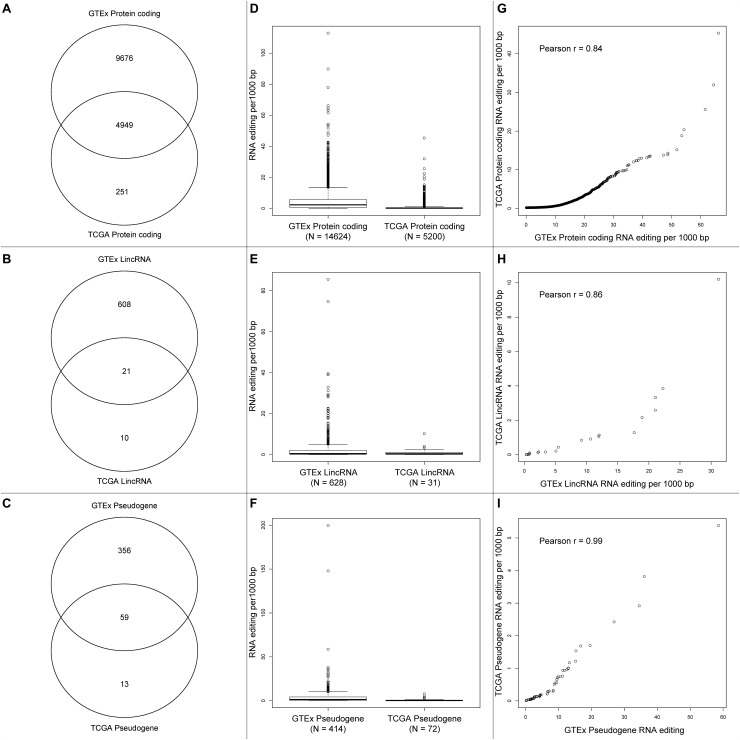
**(A–C)** Venn diagrams of protein-coding genes, lincRNA and pseudogenes with RNA editing between GTEx and TCGA. **(D–F)** Boxplots of gene-level RNA editing rates of all protein-coding genes, lincRNA, and pseudogenes between GTEx and TCGA. **(G–I)** Scatter plots of gene-level RNA editing rates of the common protein-coding genes, lincRNA, and pseudogenes between GTEx and TCGA.

We compared the RNA editing rate between GTEx and TCGA in three categories: protein coding, lincRNA, and pseudogenes. The total number of RNA editing events identified in GTEx is substantially greater than TCGA, the number of overlapped genes with RNA editing are displayed in Figure 4A–C. Because substantially higher amount of RNA editing events were identified in GTEx than TCGA, genes from GTEx had higher RNA editing rate than TCGA (Figure 4D–F). For the overlapped genes between GTEx and TCGA, the RNA editing rates were strongly correlated though clearly non-linear (Figure 4G–I), suggesting RNA editing rate are relatively consistent across these two datasets.

**FIGURE 5:**
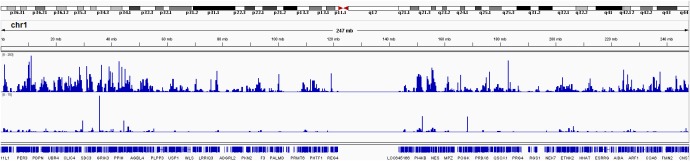
RNA editing frequency for chromosome 1 measured in both GTEx and TCGA. The RNA editing frequency is computed as number of RNA editing events observed in a 10,000 nucleotide long moving window.

Finally, we computed the genome-wide RNA editing frequency using both GTEx and TCGA data. The genome-wide RNA editing frequency was measured as number of RNA editing events observed in a moving window with the size of 10,000 nucleotides. RNA editing hotspots can be observed in both GTEx and TCGA data. However, these hotspots are most likely to be associated with gene density. For illustration purpose, the RNA editing frequency for chromosome 1 is displayed in Figure 5. The RNA editing frequencies for all chromosomes are displayed in Supplementary Figure S5.

## Discussion

Our analyses found that the RNA editing rate is highly associated with gene density, especially protein-coding gene density. Chromosome 19 was found to harbor the highest RNA editing rate among all chromosomes (excluding mitochondria). The highest RNA editing rate can be explained by the highest gene density in chromosome 19. The correlations between RNA editing rate and protein-coding gene density is higher than the correlations between RNA editing rate and lincRNA density. This could result from the bias of the source data. The TCGA RNA editing events were detected by comparing exome sequencing and RNA-seq data. Exome sequencing targets protein coding regions primarily. RNA-seq, depending on library preparation method, can target protein coding or total RNA. However, when using exome sequencing data as a reference, the detection range for RNA editing events is limited to protein-coding regions. Furthermore, the RNA editing detection is also limited to gene expression. No RNA editing can be detected with non-expressed genes. Frankly, the majority of the RNA editing source sequencing data were not designed for RNA editing detection initially but were data mined for RNA editing at a later time. The focus of protein-coding regions of the source data resulted that the current detected RNA editing events heavily reside in protein coding regions.

A majority of the RNA editing events were found in either intronic regions or 3′-UTRs for TCGA and GTEx data. This ubiquitous phenomenon has been discovered in previous findings (Alseth et al., 2014; Daniel et al., 2015). Significant proportional difference was observed for RNA editing in 3′-UTR regions between TCGA and GTEx, also suggesting potential difference of RNA editing pattern between cancerous and healthy subjects. However, such difference can also arise from a potential limitation of GTEx data. GTEx derived genomic data from tissues acquired via autopsy of donors. The GTEx inclusion criteria required tissue collection within 24 h of death. Reports (Broniscer et al., 2010; Birdsill et al., 2011) have shown that post mortem interval is negatively correlated with RNA integrity and the degradation of RNA can lead to loss of the RNA expression signal (Durrenberger et al., 2010). The effect of post-mortem interval from GTEx on RNA editing has yet to be documented.

The intronic RNA editing is less likely to cause functional consequence. The 3′UTR contains key regulatory elements such as miRNA response elements, AU-rich elements, and poly(A) tails. These elements can affect mRNA stability, export, and translation efficiency. Thus, it has been proposed that RNA editing in 3′UTR can up-regulate mRNA expression by acting as a miRNA sponge, repressing miRNA expression (Borchert et al., 2009; Wang et al., 2013; Zhang et al., 2016). It has also been suggested that RNA editing in 3′UTR can stabilize mRNA by modulating the RNA secondary structure (Brummer et al., 2017). Our across tissue type analysis of RNA editing shows that there could be tissue exclusivity of RNA editing, ∼44% of the RNA editing positions occurred in one or two types of cancer. A small portion (3.4%) of the RNA editing positions are ubiquitous across all cancer types.

There have been multiple reports that RNA editing events tend to occur in close vicinity (Carmi et al., 2011; Danecek et al., 2012; Porath et al., 2014; John et al., 2017), these phenomena have been referred to as clusters, editing islands or hyper-editing regions. We found many of the protein-coding genes had high RNA editing rates after adjusting for the gene length, many of these RNA editing causes non-synonymous changes, which has been proven to generate alternative protein sequences (Peng et al., 2018) which can potentially significantly alter function, including drug sensitivity (Han et al., 2015). Notably, 9 out of the top 10 RNA editing protein coding genes from TCGA had prognostic value (Table 2). However, we cannot assert that the RNA editing modified gene expression. The exact mechanisms and potential functions of RNA editing in these protein coding genes remain to be discovered.

A portion of the RNA editing events do occur in non-coding RNAs, which have gained substantial interest in recent years. One of the common types of non-coding RNA that we explored in our study is lincRNA, which are traditionally thought to be non-functional. The increasing reports of lincRNA-disease associations over the last decades suggest that there are unknown mechanisms by which lincRNA exerts its influence (Wang et al., 2017). There have been suggestions that lincRNAs function through regulation of protein coding genes by trans expression quantitative trait loci (eQTL) (Cabili et al., 2011; Hon et al., 2017). Another type of non-coding RNA we examined was the pseudogenes. The known potential primary function of pseudogenes involve them acting as miRNA sponges, alleviating the regulation effect of miRNAs (Poliseno et al., 2010; Karreth et al., 2015). Our results show that lincRNAs and pseudogenes are among the top hyper RNA edited genes in GTEx. Whether RNA editing affects the potential functions of lincRNA and pseudogene has been understudied and will require further exploration.

## Ethics Statement

This study uses publically available data downloaded from GDC data repository. It does not contain any patient information or patient data.

## Author Contributions

MC, YG, DS, and Y-yZ wrote the manuscript. MC, HY, and WY performed the analyses and designed the figures and tables for the first submission. YG designed the study. SN, QS, and OO performed the additional analyses and designed the new figures during revisions.

## Conflict of Interest Statement

The authors declare that the research was conducted in the absence of any commercial or financial relationships that could be construed as a potential conflict of interest.

## Supplementary Material

FIGURE S1 Histogram of shared RNA editing sites across multiple cancers filtered by four different average RNA editing level: A > 0%, B > 10%, C > 25%, D > 50%. Majority of the RNA editing sites occurs only in 1 or 2 cancers.

FIGURE S2 Scatter plot between number of RNA editing found and sample size in the 14 TCGA cancer types. No correlation was observed.

FIGURE S3 Pie charts to display the distribution of RNA editing events by genomic location categories by each cancer type.

FIGURE S4 Manhattan plots that depicting the genome-wide results of RNA editing level difference between tumor and normal samples in TCGA.

FIGURE S5 Genome-wide RNA editing frequency for all chromosomes.

TABLE S1 All RNA editing positions across the 14 cancer types examined.

TABLE S2 The number of RNA editing events detected in both GTEx and TCGA.

TABLE S3 Differential RNA editing results.

TABLE S4 Functional analysis results from DAVID.

TABLE S5 Common functional categories shared by cancers from genes with RNA editing events.

TABLE S6 Top 10 hyper editing genes by cancer.
