# Corrigendum: Genomic Positional Dissection of RNA Editomes in Tumor and Normal Samples

**DOI:** 10.3389/fgene.2020.00162

**Published:** 2020-02-25

**Authors:** Michael Chigaev, Hui Yu, David C. Samuels, Quanhu Sheng, Olufunmilola Oyebamiji, Scott Ness, Wei Yue, Ying-yong Zhao, Yan Guo

**Affiliations:** ^1^Department of Internal Medicine, The University of New Mexico, Albuquerque, NM, United States; ^2^Department of Molecular Physiology and Biophysics, Vanderbilt Genetics Institute, Vanderbilt University School of Medicine, Nashville, TN, United States; ^3^Department of Biostatistics, Vanderbilt University Medical Center, Nashville, TN, United States; ^4^Key Laboratory of Resource Biology and Biotechnology in Western China, School of Life Sciences, Northwest University, Xi'an, China

**Keywords:** RNA editing, A-to-I, adenosine to inosine, cancer, non-coding RNAs, TCGA, GTEx

In the published article, there was an error in affiliation for the last author Yan Guo. Yan Guo should only be associated with affiliation #1 (University of New Mexico) instead of both #1 and #4.

The authors apologize for this error and state that this does not change the scientific conclusions of the article in any way. The original article has been updated.

