# Supplementary figures and images for: Genomic Positional Dissection of RNA Editomes in Tumor and Normal Samples
Source: Front Genet. 2019 Mar 20;10:211. doi: 10.3389/fgene.2019.00211 (PMC6435843; doi:10.3389/fgene.2019.00211)

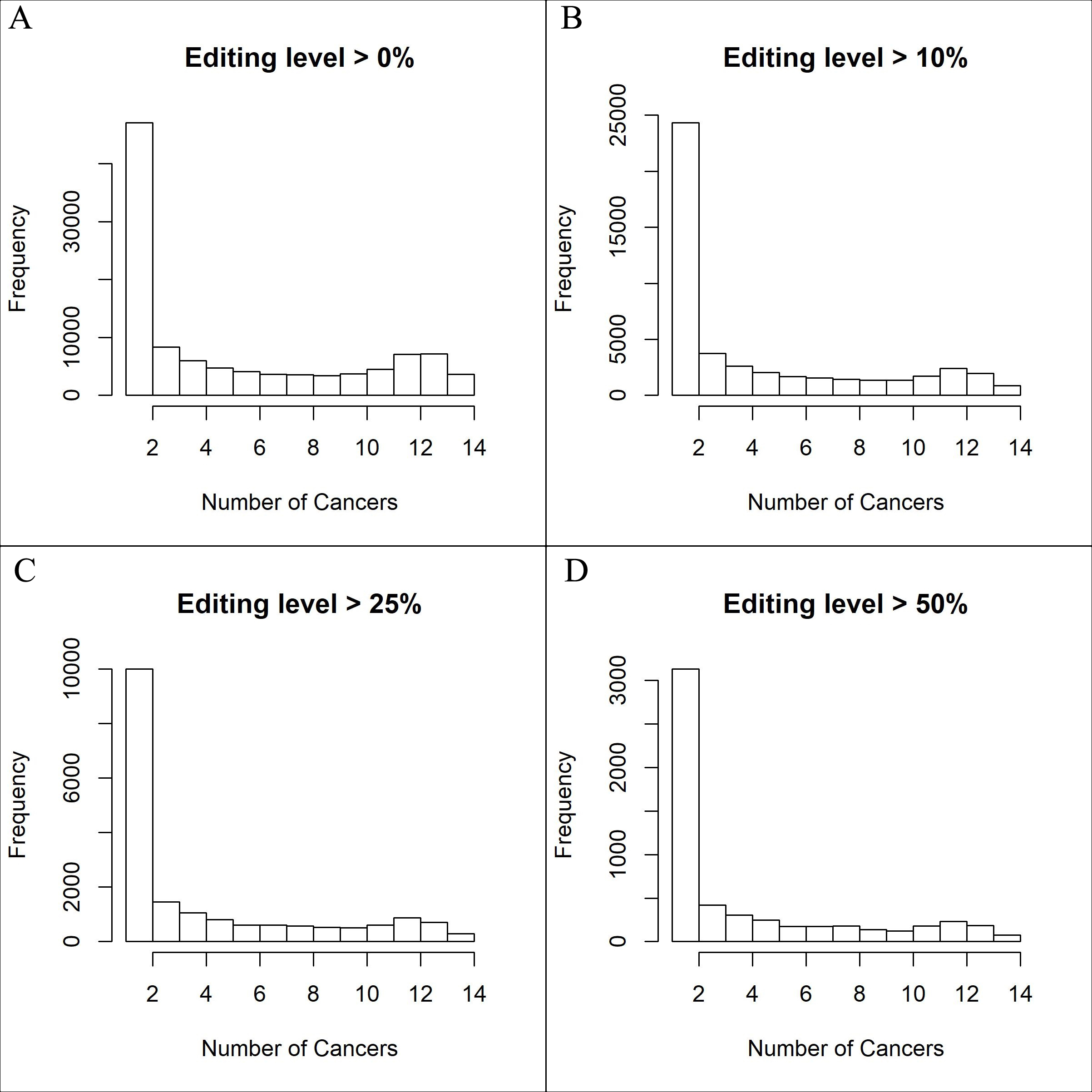

Supplement: FIGURE S1 — Histogram of shared RNA editing sites across multiple cancers filtered by four different average RNA editing level: A > 0%, B > 10%, C > 25%, D > 50%. Majority of the RNA editing sites occurs only in 1 or 2 cancers. [file Data_Sheet_1.ZIP › FigureS1_hist_by_editing_level.jpg]

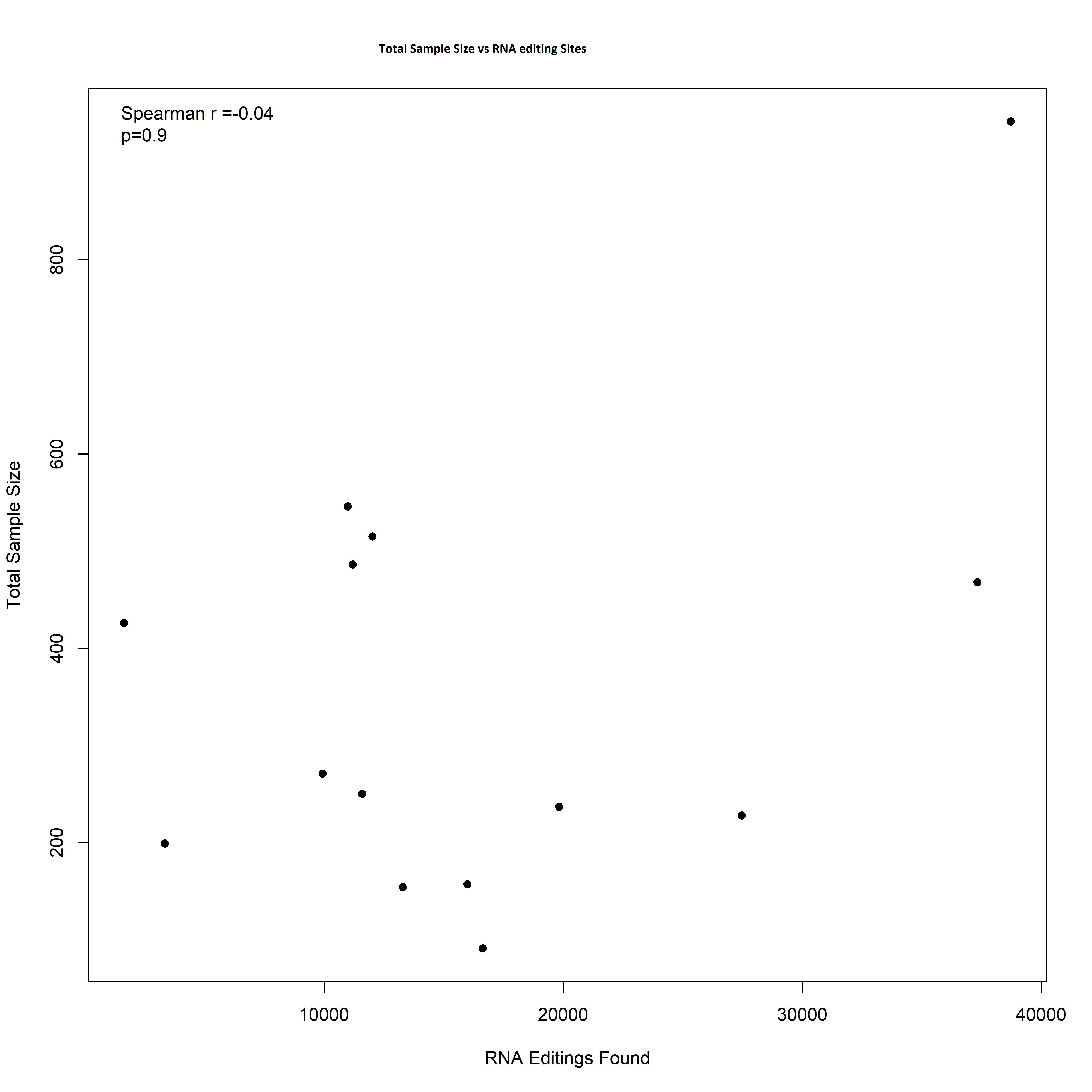

Supplement: FIGURE S1 — Histogram of shared RNA editing sites across multiple cancers filtered by four different average RNA editing level: A > 0%, B > 10%, C > 25%, D > 50%. Majority of the RNA editing sites occurs only in 1 or 2 cancers. [file Data_Sheet_1.ZIP › FigureS2.jpg]

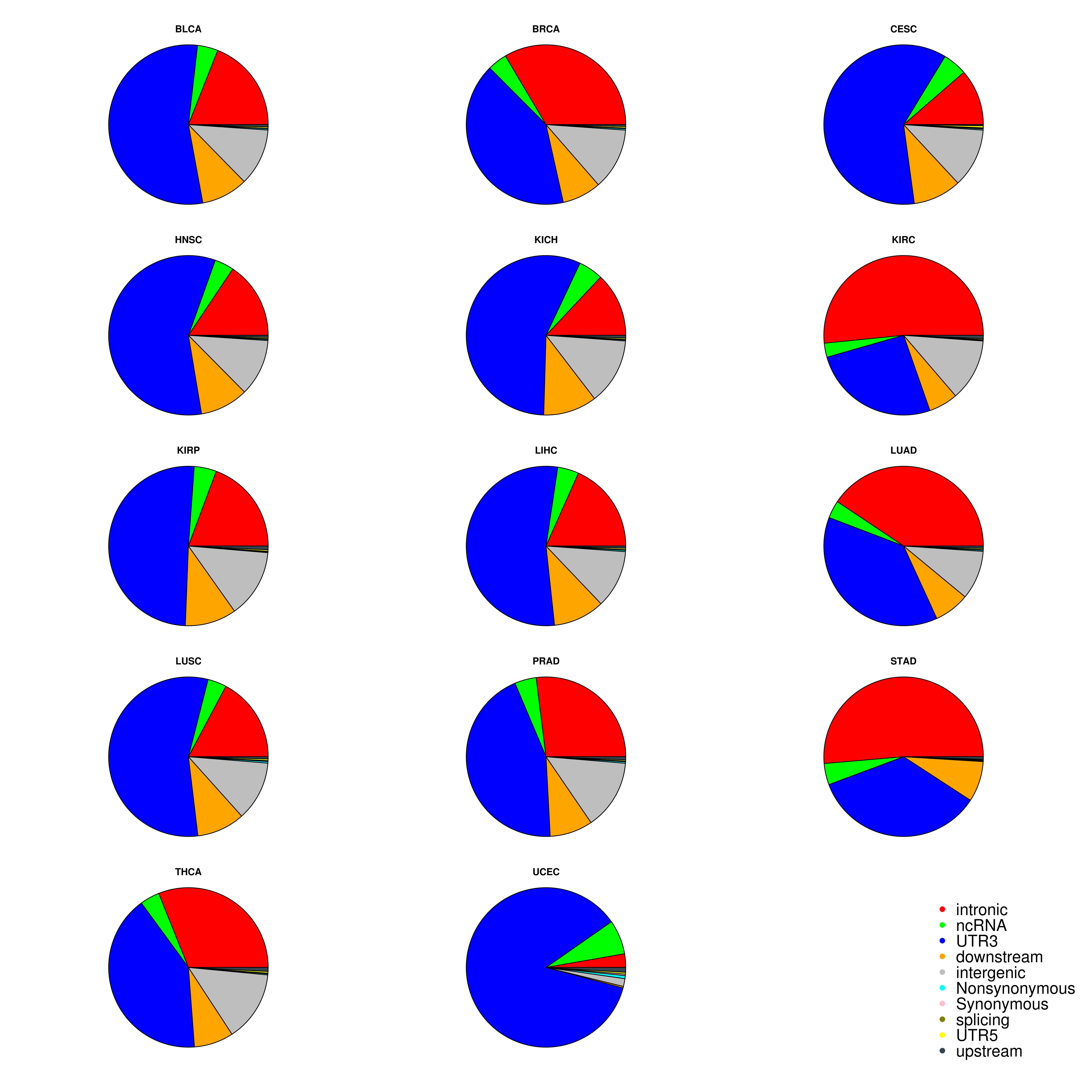

Supplement: FIGURE S1 — Histogram of shared RNA editing sites across multiple cancers filtered by four different average RNA editing level: A > 0%, B > 10%, C > 25%, D > 50%. Majority of the RNA editing sites occurs only in 1 or 2 cancers. [file Data_Sheet_1.ZIP › FigureS3_pieChartSideBySide.jpg]

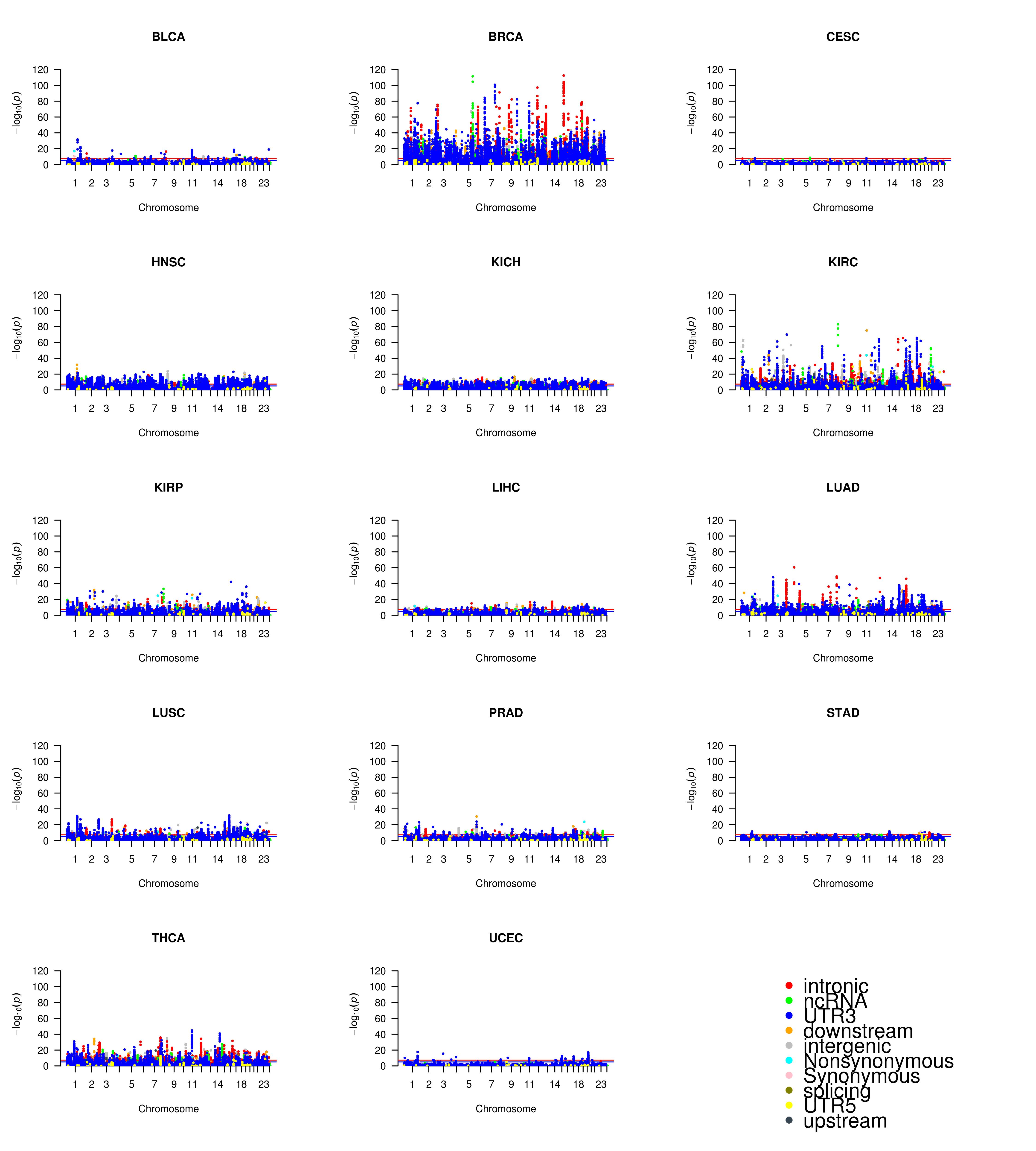

Supplement: FIGURE S1 — Histogram of shared RNA editing sites across multiple cancers filtered by four different average RNA editing level: A > 0%, B > 10%, C > 25%, D > 50%. Majority of the RNA editing sites occurs only in 1 or 2 cancers. [file Data_Sheet_1.ZIP › FigureS4_manhattan.jpg]

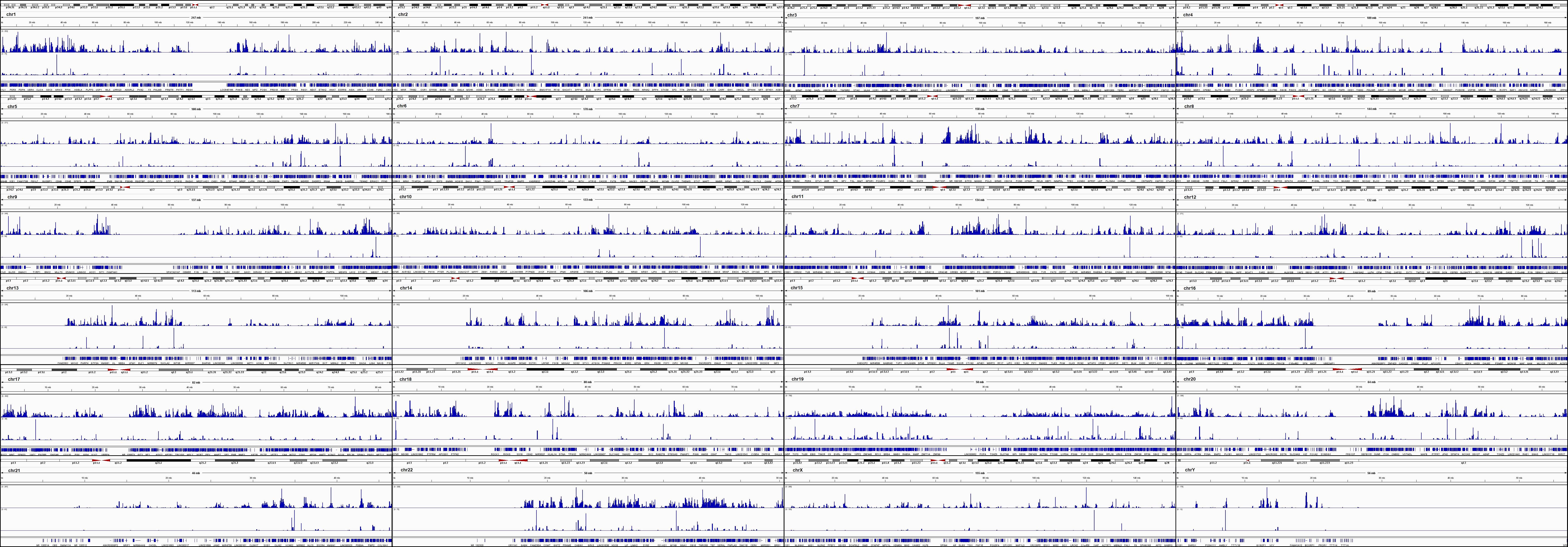

Supplement: FIGURE S1 — Histogram of shared RNA editing sites across multiple cancers filtered by four different average RNA editing level: A > 0%, B > 10%, C > 25%, D > 50%. Majority of the RNA editing sites occurs only in 1 or 2 cancers. [file Data_Sheet_1.ZIP › FigureS5.jpg]
